# Dual-Task Elderly Gait of Prospective Fallers and Non-Fallers: A Wearable-Sensor Based Analysis

**DOI:** 10.3390/s18041275

**Published:** 2018-04-21

**Authors:** Jennifer Howcroft, Edward D. Lemaire, Jonathan Kofman, William E. McIlroy

**Affiliations:** 1 Department of Systems Design Engineering, University of Waterloo, Waterloo, ON N2L 3G1, Canada; jirwin02@gmail.com; 2 Centre for Rehabilitation Research and Development, Ottawa Hospital Research Institute, Ottawa, ON K1H 8M2, Canada; elemaire@ohri.ca; 3 Faculty of Medicine, University of Ottawa, Ottawa, ON K1H 8M5, Canada; 4 Department of Kinesiology, University of Waterloo, Waterloo, ON N2L 3G1, Canada; wmcilroy@uwaterloo.ca

**Keywords:** wearable sensors, plantar pressure, accelerometer, dynamic stability, dual task gait, elderly, older adults

## Abstract

Wearable sensors could facilitate point of care, clinically feasible assessments of dynamic stability and associated fall risk through an assessment of single-task (ST) and dual-task (DT) walking. This study investigated gait changes between ST and DT walking and between older adult prospective fallers and non-fallers. The results were compared to a study based on retrospective fall occurrence. Seventy-five individuals (75.2 ± 6.6 years; 47 non-fallers, 28 fallers; 6 month prospective fall occurrence) walked 7.62 m under ST and DT conditions while wearing pressure-sensing insoles and accelerometers at the head, pelvis, and on both shanks. DT-induced gait changes included changes in temporal measures, centre of pressure (CoP) path stance deviations and coefficient of variation, acceleration descriptive statistics, Fast Fourier Transform (FFT) first quartile, ratio of even to odd harmonics, and maximum Lyapunov exponent. Compared to non-fallers, prospective fallers had significantly lower DT anterior–posterior CoP path stance coefficient of variation, DT head anterior–posterior FFT first quartile, ST left shank medial–lateral FFT first quartile, and ST right shank superior maximum acceleration. DT-induced gait changes were consistent regardless of faller status or when the fall occurred (retrospective or prospective). Gait differences between fallers and non-fallers were dependent on retrospective or prospective faller identification.

## 1. Introduction

For elderly adults, dual-task walking can reveal impaired executive function and mobility control problems, which can relate to fall risk [1,2,3]. Biomechanically, dynamic stability can be affected during dual-task (DT) walking since an individual must control centre of mass displacements with a changing base of support [4] using sensorimotor and cognitive processes [5], particularly executive function and attention [6], while accomplishing a second attention-demanding task.

Our previous work based on retrospective fall occurrence and other retrospective-fall based studies have shown that wearable sensors can provide useful information for understanding dynamic stability under DT gait conditions, thereby assisting in fall risk identification. Wearable sensors are feasible for integration into point-of-care health assessments, facilitating timely and quantitative fall risk assessments. DT gait can affect temporal parameters [1,2,3,7,8,9,10,11,12], variability [1,2,9,12], and stability [1,12], and produce more missteps [13] compared to single-task (ST) gait.

Studies of DT for predicting falls [14,15] are inconclusive. DT measures that differentiate elderly fallers from non-fallers include lower gait speed [16,17,18,19]; greater swing time [9,20,21] and stride time [22] variability; greater head and pelvis variability [12]; lower pelvis stability [12]; and greater DT cost (difference between single and dual task performance) for mean step width, step time, and step length variability [23]. However, other studies did not improve fall prediction by adding a second task [24,25]. Some of these studies identified differences between elderly fallers and non-fallers based on retrospective fall occurrence, where falls occurred before the study data collection session, while others were based on prospective fall occurrence for which falls occurred after the study data collection session.

Retrospective fall occurrence has limitations of inaccurate recall of falls and gait pattern changes after the fall in an attempt to increase stability [26]. It is currently unclear whether similar gait differences occur between elderly fallers and non-fallers regardless of whether retrospective or prospective fall occurrence is used and whether this choice of methodology has an impact on identified gait differences. One study [27] did examine accelerometer-derived gait variables in an at-home environment and found similar odds ratios for retrospective and prospective falls.

This study examined gait patterns for differences associated with walking condition (ST, DT) and prospectively identified faller status based on a clinically feasible test using wearable sensors, and the twenty-five foot (7.62 m) walk [28,29] that could be applied at the point of care. This study also investigated the retrospective and prospective fall methodologies for elderly dual-task gait assessment of fall risk. Therefore, the objectives of this study were to: (1) detect differences between prospectively identified older adult fallers and non-fallers using plantar pressure and body acceleration gait measures, (2) identify DT-induced gait changes, and (3) compare retrospective and prospective fall methodologies by comparing this study’s results based on prospective fall occurrence to our earlier work [12] based on retrospective fall occurrence.

## 2. Materials and Methods

### 2.1. Participants

A convenience sample of 76 older adults, aged 65 years or older and without a fall in the six months before evaluation, were recruited from the community. Participants were excluded if they had a cognitive disorder (self-reported) or were unable to walk for six minutes without an assistive device. Three participants used a cane (one faller, two non-fallers) and two used a walker (both fallers); however, these devices were not used during walking assessments. Faller group criterion was at least one fall during the six-month follow-up period. A fall was defined as an event that results in a person coming to rest unintentionally on the ground or other lower level, excluding falls due to a stroke or overwhelming hazard [30]. One participant did not complete the six-month follow-up, leaving 75 participants: 47 non-fallers (17 male, 30 female, 75.3 ± 5.5 years old, height: 164.8 ± 10.5 cm, weight: 73.3 ± 13.6 kg) and 28 fallers (14 male, 14 female, 75.0 ± 8.2 years old, height: 165.7 ± 9.3 cm, weight: 73.4 ± 13.2 kg). Prospective falls per person ranged from one to four (average = 1.3). Data from this participant group were also used in other research on prospective fall occurrence [31,32,33]. The University of Waterloo Research Ethics Committee approved the study, and all participants gave informed written consent.

### 2.2. Protocol

A complete description of the data collection protocol is given in [12]. Briefly, participants wore F-Scan pressure-sensing insoles (F-Scan 3000E, Tekscan, Boston, MA, USA) in their shoes and tri-axial accelerometers (X16-1C, Gulf Coast Data Concepts, Waveland, MS, USA) attached to bands on the posterior head and the lateral shanks just above the ankle, and to a belt at the posterior pelvis. Accelerometer and plantar pressure data were collected while participants walked 7.62 m with (DT) and without (single-task, ST) a verbal-task cognitive load, in separate trials. The task was the verbal fluency test that involves saying as many words as possible that start with the letters A, F, or S [34]. Accelerometer measurement range was ±16 g and data were collected at 50 Hz. Plantar pressure data were collected at 120 Hz and the pressure measurement range was zero to 517 kPa.

After the walking session, participants recorded fall occurrence for the following six months using a calendar and fall information form. Participants were contacted monthly to collect fall information.

### 2.3. Data Processing

For the ST and DT trials, the following plantar pressure parameters were calculated: number, length, and duration of medial–lateral (ML) and posterior deviations per stance; anterior–posterior (AP) and ML stance phase centre of pressure (CoP) path coefficients of variation (CoV); CoP path velocity; cadence; stride time; stance time; swing time; percent stance time; percent double support time; stride time symmetry index; stride time, stance time, and swing time CoV; and impulse parameters (I1: foot-strike to first peak, I2: first peak to minimum, I3: minimum to second peak, I4: second peak to foot-off, I5: foot-strike to minimum, I6: minimum to foot-off, and I7; foot-strike to foot-off) [12]. Deviations were unexpected movements in the CoP path. Since the CoP path should advance monotonically and anteriorly, any posterior CoP movements were identified as posterior deviations (PD). Similarly, since ML CoP movements should be relatively smooth, CoP ML path movements exceeding a dual threshold of ±0.5 mm/frame were identified as deviations.

In addition, the following accelerometer parameters were calculated: maximum, mean, and standard deviation of acceleration for superior, inferior, anterior, posterior, right, and left axes, and ratio of even to odd harmonics (REOH); Fast Fourier Transform (FFT) first quartile; and maximum Lyapunov exponent (MLE) for vertical, AP, and ML axes [12].

### 2.4. Data Analysis

Data analysis was performed as in [12], allowing a direct comparison to statistical results based on retrospective faller classification. Briefly, mixed-design ANOVA tests (Supplementary Tables S1–S5) were performed for each sensor with a 2-factor within-subject walking condition (ST, DT) and a 2-factor between-subject faller status condition (faller, non-faller). The critical *p*-value for all comparisons was 0.05. Post-hoc assessments were performed for variables with a significant main effect for walking or faller conditions or a significant interaction effect. For post-hoc assessments, normality was assessed with the Shapiro–Wilk Test and variance was assessed with the Levene’s Test. Wilcoxon Signed-Rank tests were used to compare ST and DT walking conditions for non-normal datasets and paired *t*-tests were used for normal datasets. Faller and non-faller comparisons used Mann–Whitney U tests for non-normal data, Welch’s *t*-tests for normal and unequal variance data, and independent *t*-tests for normal and equal variance data. Corrections for multiple tests were applied [35]; thus, not all variables with *p* < 0.05 were significantly different.

## 3. Results

Results for faller and non-faller groups based on prospective fall occurrence are presented in this paper. Mixed-design ANOVA results for each sensor are presented in Supplementary Tables S1–S5. Results for faller and non-faller groups based on retrospective fall occurrence were presented in our earlier study [12].

### 3.1. Gait Velocity

#### 3.1.1. Differences between Walking Conditions

For fallers, DT gait velocity (0.95 ± 0.21 m/s) was significantly lower (*p* < 0.001) than ST (1.17 ± 0.16 m/s). For non-fallers, DT gait velocity (0.95 ± 0.23 m/s) was also significantly lower (*p* < 0.001) than ST (1.22 ± 0.23 m/s).

#### 3.1.2. Differences between Prospective Fallers and Non-Fallers

No significant differences were found between fallers and non-fallers for ST or DT gait velocity (*p* ≥ 0.261).

### 3.2. Pressure-Sensing Insole Measures

**Figure 1:**
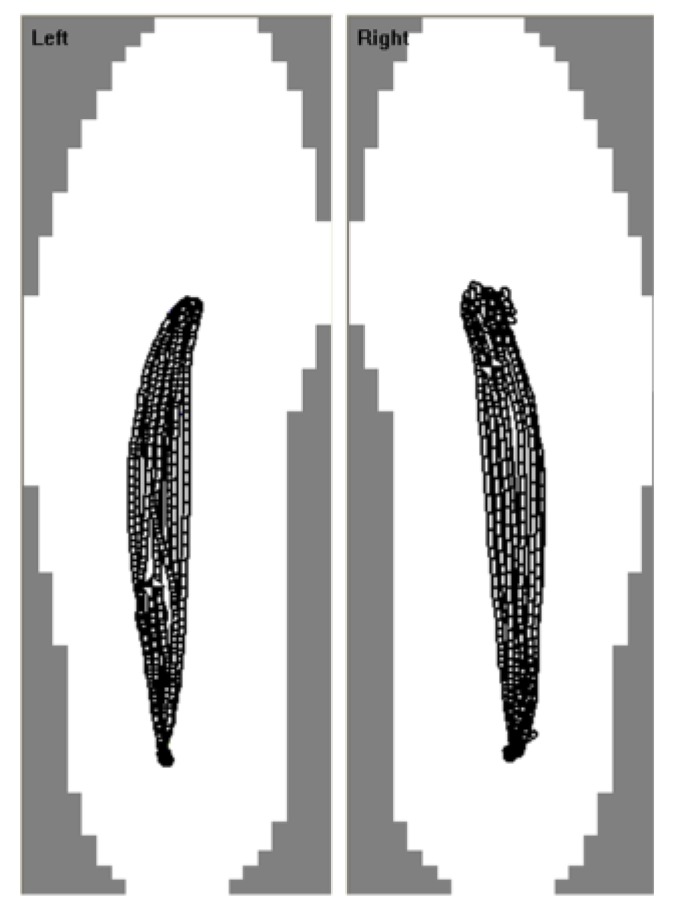
Typical plantar pressure derived centre of pressure paths for ST gait [36].

An example of a typical CoP path for 10 strides under ST conditions for the left and right feet of one participant is shown in Figure 1.

#### 3.2.1. Differences between Walking Conditions

For fallers, DT parameters were significantly greater than ST for PD per stride, ML deviation duration, stride time, stance time, swing time, stride time CoV, stride time symmetry index, I1, I4, I6, and I7 (Table 1). DT parameters were significantly lower than ST for minimum, mean, median CoP velocity; cadence; and I2.

For non-fallers, DT parameters were significantly greater than ST for PD per stride, ML deviation duration, stride time, stance time, swing time, stride time CoV, stride time symmetry index, AP and ML CoV, I1, I4, I5, I6, and I7 (Table 1). DT parameters were significantly lower than ST for minimum, mean, and median CoP velocity; and cadence.

#### 3.2.2. Differences between Prospective Fallers and Non-Fallers

For DT gait, fallers had significantly lower AP CoV than non-fallers (*p* = 0.046). No significant differences were found between fallers and non-fallers for ST gait.

### 3.3. Accelerometer Measures

**Figure 2:**
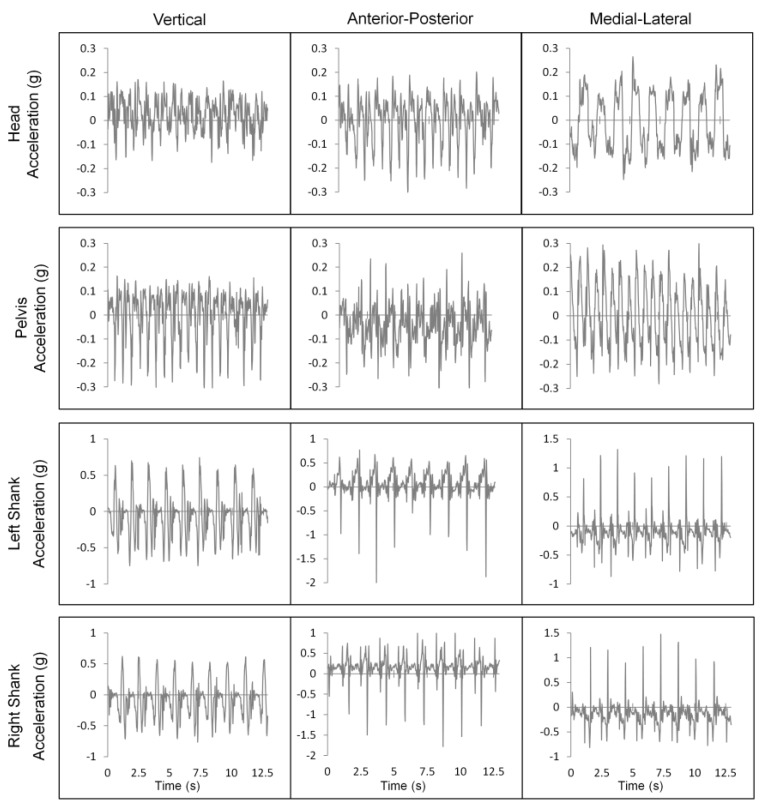
Typical accelerometer signals for ST gait for all accelerometer locations: head, posterior pelvis, left shank, and right shank. For the vertical axis, positive is upwards/superior; for the AP axis, positive is anterior; for the ML axis, positive is toward the participant’s right [36].

Examples of typical accelerometer signals under ST conditions for one participant are shown in Figure 2.

#### 3.3.1. Differences between Walking Conditions

For fallers and non-fallers, significant differences were found between ST and DT gait conditions (Table 2, Table 3, Table 4 and Table 5). For fallers, the following acceleration variables were significantly lower for DT compared to ST:

- Head, right shank, left shank
  ○ AP: FFT first quartile
- Pelvis, right shank, left shank
  ○ inferior: mean; anterior: maximum, mean, SD; left: mean, SD
- Head, right shank
  ○ V: FFT first quartile
- Pelvis, right shank
  ○ right: SD
- Pelvis, left shank
  ○ superior: mean; posterior: mean, SD
- Right shank, left shank
  ○ inferior: maximum, SD
- Pelvis
  ○ posterior: maximum
- Right shank
  ○ ML: FFT first quartile; right: maximum, mean; left: maximum
- Left shank
  ○ AP, ML: MLE; superior: maximum, SD.

Superior maximum, mean, and SD of the head were significantly greater for DT than ST. For non-fallers, the following variables were significantly lower for DT compared to ST gait:

- Head, pelvis, left shank, right shank
  ○ V: FFT first quartile; anterior: mean
- Head, right shank, left shank
  ○ AP: FFT first quartile
- Pelvis, right shank, left shank
  ○ ML: FFT first quartile; superior: maximum, mean, SD; inferior: maximum, mean, SD; anterior: maximum, SD; posterior: mean; right: mean, SD; left: maximum, mean, SD
- Pelvis, right shank
  ○ right: maximum
- Pelvis, left shank
  ○ ML: MLE
- Pelvis
  ○ AP: REOH; posterior: maximum, SD

The superior maximum, mean, and SD and right maximum, mean, and SD of the head were significantly greater for DT compared to ST gait.

#### 3.3.2. Differences between Prospective Fallers and Non-Fallers

Significant differences were found in some accelerometer measures between fallers and non-fallers. For the head accelerometer, the AP FFT first quartile was significantly lower (*p* = 0.011) for fallers than non-fallers for DT gait. For the left shank accelerometer, the ML FFT first quartile was significantly lower (*p* = 0.045) for fallers than non-fallers for ST gait. For the right shank accelerometer, the superior maximum acceleration was significantly lower (*p* = 0.041) for fallers than non-fallers for ST gait.

## 4. Discussion

Differences between ST and DT gait and between prospective fallers and non-fallers were identified from a clinically feasible 25 ft (7.62 m) walking assessment, using wearable accelerometers and pressure-sensing insoles that could be implemented as a point-of-care fall risk assessment. Measures relevant to DT-induced gait changes for prospective faller and non-faller groups were similar to DT-induced gait changes for retrospective faller and non-faller groups. These measures were associated with impulse, movement frequency, abnormal foot movements, and body segment accelerations. However, measures that differentiated between prospective fallers and non-fallers (stance path CoV, proportion of low frequency acceleration signals (FFT first quartile)) were not the same as measures that differentiated retrospective fallers and non-fallers (head posterior standard deviation of acceleration, REOH, MLE).

### 4.1. Gait Differences between Fallers and Non-Fallers

Gait differences were identified between prospective fallers and non-fallers using measures derived from the head, shank accelerometers, and pressure sensing insoles. Prospective fallers had smaller AP and ML FFT first quartile frequencies at the head during DT and at the left shank during ST, respectively. Less low frequency content should indicate more numerous higher-frequency gait perturbations. FFT findings suggest that fallers exhibited dynamic stability issues related to high frequency gait perturbations with DT that may have increased fall risk [33]. However, AP CoV during DT was lower for prospective fallers than non-fallers, suggesting decreased faller CoP stance path variability at the foot–shoe interface. Prospective fallers also had lower maximum superior acceleration at the right shank during ST, which could indicate a reduced magnitude of acceleration near the foot–shoe interface. The dichotomy exhibited in prospective fallers of dynamic stability issues in head and left shank movements and lower variability at the foot–shoe interface, compared to non-fallers, did not occur for retrospective fallers. For retrospective fall occurrence, measures with a significant difference (head posterior standard deviation, posterior pelvis AP REOH, posterior pelvis vertical MLE) between non-fallers and fallers indicated increased variability and decreased stability [12]. Therefore, research with prospective faller data is important when assessing gait differences using measures derived from wearable accelerometers and insoles.

### 4.2. Temporally Related DT-Induced Gait Differences

Gait velocity, cadence, and all CoP stance velocity measures, except maximum CoP stance velocity, decreased with DT. Stride time, stance time, and swing time increased. These temporal parameter results agree with our retrospective study [12] and previously published results for retrospective fallers and non-fallers [1,2,3,7,8,9]. Our study’s swing time results were similar to Wild et al. [10], but not Hausdorff et al. [2] and Springer et al. [9]. Body weight normalized impulse increased with a cognitive load for all gait phases except I2 (first peak to minimum) and I3 (minimum to second peak). Since stance time increases (fallers: 14%, non-fallers: 17%) were greater than overall impulse increases (fallers: 9%, non-fallers: 15%), stance time was likely the main contributor to increased impulse during DT. These temporal and impulse changes may be part of a compensatory, conservative gait strategy to maintain dynamic stability. Furthermore, these temporally-related DT gait differences occurred regardless of fall status (faller, non-faller) or fall occurrence (retrospective versus prospective). Therefore, gait changes to maintain dynamic stability in response to DT were not dependent on fall risk or fall occurrence.

### 4.3. Variability and Stability Related DT-Induced Gait Differences

Several of the DT-induced gait differences seem to be related to increased variability and decreased stability during DT gait. For DT, the number of posterior CoP path deviations and duration of ML CoP path deviations increased, for both prospective fallers and non-fallers. For pressure-sensor derived variables, increased DT variability was identified by increased stride time CoV, increased stride time symmetry index, and increased AP and ML CoV (non-fallers only) in our current, prospective fall occurrence study. Increased DT variability was also identified from decreased vertical (head and right shank for fallers; all accelerometer locations for non-fallers), AP (head, right shank, left shank for fallers and non-fallers), and ML (right shank for fallers; pelvis, right shank, and left shank for non-fallers) FFT first quartile frequencies; and AP REOH (posterior pelvis for non-fallers). Decreased FFT first quartile frequency indicated less low frequency content with a cognitive load. Decreased REOH indicated that a smaller proportion of the acceleration signal was in phase with the participant’s stride frequency, indicating increased gait variability.

Similar to the temporally-related DT-induced gait differences, most of the variability and stability related DT-induced gait differences occurred regardless of fall status or fall occurrence (retrospective versus prospective). The findings of more numerous posterior CoP path deviations, increased duration of ML CoP deviations, greater stance path CoV in non-fallers, and increased stride time CoV are in line with findings reported in our retrospective study [12]. Similarly, instances of decreased REOH and decreased FFT first quartile frequencies were found in our retrospective study [12]. Therefore, increased gait variability and CoP path deviations are consistent markers of decreased walking stability under DT gait conditions.

DT acceleration maximum, mean, and SD decreased along all axes for all accelerometer locations, compared to ST, with only the head location having instances of increased acceleration. During DT walking, increased head accelerations in the superior (fallers and non-fallers) and right (non-fallers) axes may be from non-gait related movements during particularly attention-demanding periods (e.g., struggling to think of another word, researcher prompts to continue with cognitive task). Decreased acceleration SD at the pelvis and shanks indicated decreased variability with a cognitive load, indicating a conservative stiffening strategy where body motions are reduced to minimize centre of mass deviations [37]. Decreased acceleration SD occurred under DT conditions for both retrospective [12] and prospective fallers and non-fallers. Decreased acceleration SD occurred more frequently for non-fallers than fallers. This may indicate that non-fallers are better than fallers at compensating for increased DT demands by reducing acceleration variability. The acceleration SD measure may be useful for identifying compensatory strategies in non-fallers and should be a focus of future investigations.

### 4.4. Limitations

The focus of this study was on wearable-sensor derived measures. As such, cognitive task performance was not measured. Cognitive and gait prioritization inconsistencies could not be assessed and may have increased inter-individual variability. Individuals tend to prioritize motor tasks over cognitive tasks in DT scenarios [10], but prioritization across participants can vary, masking faller and non-faller gait differences [6]. To reduce prioritization effects, participants were encouraged to continue with the cognitive task when they struggled or stopped listing words, thus preventing cognitive task abandonment. Future studies should assess cognitive task performance during DT assessments. Single-task cognitive performance could be evaluated to determine the cognitive dual-task cost.

This study examined features derived from a 7.62 m (25 ft) walking trial. This distance translates to clinical settings where the “25-Foot Walk Test” [28,29] could be performed; however, a longer walking trial may be more reflective of everyday walking for older adults. The 7.62 m walking distance, which elicited 7.6 ± 1.5 strides for ST and 8.6 ± 2.0 strides for DT, may have affected MLE reliability, since stable MLE measures occurred after 35 strides in [38].

While a correction for multiple comparisons was performed, a large number of variables were considered in this analysis, which increases the potential for Type 1 errors.

## 5. Conclusions

Differences between ST and DT gait and between prospective fallers and non-fallers were identified from wearable-sensor based gait data during a short walking trial, which is feasible as a point-of-care fall risk assessment. DT-induced gait changes were consistent regardless of faller status or fall occurrence (retrospective or prospective). Some DT-induced gait changes appeared to indicate increased variability and decreased dynamic stability under DT conditions while other changes may represent elements of a conservative, compensatory gait strategy aimed at minimizing the influence of DT-induced dynamic stability alterations. Therefore, point-of-care assessments should focus on identifying gait changes related to decreased dynamic stability, which indicate a worsened gait pattern. Identified compensatory strategies may offset the impact of decreased dynamic stability and require further investigation to determine their effectiveness. Differences between prospective fallers and non-fallers were related to variability and the proportion of low frequency acceleration signals during ST and DT gait. Prospective fallers exhibited greater variability in head and left shank movements but lower variability at the foot–shoe interface than prospective non-fallers. Some gait differences between fallers and non-fallers were dependent on whether fallers were identified based on retrospective and prospective fall occurrence. Therefore, measures related to fall risk, based on prospective fall occurrence, are more likely to succeed as part of a clinical, point-of-care fall risk assessment protocol than measures based on retrospective fall occurrence. Research with prospective faller data is important when assessing gait differences using measures derived from wearable accelerometers and instrumented insoles and clinically feasible assessments to ensure their applicability for point-of-care fall risk assessments.

## Figures and Tables

**Table 1:** Means and SD for pressure-sensing insole variables with a significant (*p* < 0.05) mixed-design ANOVA result (Supplementary Table S1). Bold *p*-values indicate a significant difference between single-task (ST) and dual-task (DT) conditions after correction for multiple comparisons.

	Fallers			Non-Fallers		
	ST	DT	*p*	ST	DT	*p*
**CoP Path**						
PD per Stride	1.8 ± 2.7	2.6 ± 3.1	**<0.001**	1.5 ± 2.0	2.5 ± 2.1	**<0.001**
Lateral Deviation Length (mm)	0.9 ± 0.6	1.1 ± 0.7	0.165	1.0 ± 1.2	1.4 ± 1.4	0.051
Medial–Lateral (ML) Deviation Duration (s)	0.029 ± 0.013	0.038 ± 0.014	**0.029**	0.031 ± 0.015	0.037 ± 0.017	**0.019**
Min Centre of Pressure (CoP) Velocity (m/s)	0.028 ± 0.010	0.021 ± 0.009	**0.001**	0.031 ± 0.012	0.023 ± 0.010	**<0.001**
Mean CoP Velocity (m/s)	0.284 ± 0.038	0.249 ± 0.044	**<0.001**	0.293 ± 0.048	0.250 ± 0.049	**<0.001**
Median CoP Velocity (m/s)	0.247 ± 0.034	0.208 ± 0.035	**<0.001**	0.250 ± 0.041	0.213 ± 0.047	**<0.001**
**Temporal**						
Cadence (steps/minute)	109.6 ± 10.0	98.4 ± 12.9	**<0.001**	111.9 ± 10.5	96.4 ± 14.9	**<0.001**
Stride Time (s)	1.11 ± 0.10	1.24 ± 0.18	**<0.001**	1.09 ± 0.11	1.28 ± 0.21	**<0.001**
Stance Time (s)	0.73 ± 0.09	0.83 ± 0.13	**<0.001**	0.72 ± 0.09	0.84 ± 0.15	**<0.001**
Swing Time (s)	0.38 ± 0.05	0.42 ± 0.07	**<0.001**	0.37 ± 0.06	0.44 ± 0.07	**<0.001**
Stride Time CoV	0.03 ± 0.03	0.04 ± 0.02	**0.031**	0.03 ± 0.01	0.04 ± 0.02	**<0.001**
Stride Time Symmetry Index	2.13 ± 1.14	2.95 ± 1.79	**0.005**	2.18 ± 1.41	2.86 ± 1.50	**0.026**
**CoP Path Stance Phase CoV**						
CoV Anterior–Posterior (AP)	4.90 ± 1.63	5.22 ± 1.42	0.248	4.48 ± 1.54	6.17 ± 2.21	**<0.001**
CoV ML	6.57 ± 2.44	7.39 ± 2.60	0.059	6.66 ± 2.33	7.70 ± 2.96	**0.007**
**Impulse (Ns/kg)**						
Foot-strike to first peak (I1)	1.22 ± 0.41	1.40 ± 0.52	**0.009**	1.20 ± 0.50	1.50 ± 0.66	**<0.001**
First peak to min (I2)	1.22 ± 0.48	1.10 ± 0.49	**0.004**	1.27 ± 0.49	1.24 ± 0.51	0.435
Min to second peak (I3)	1.83 ± 0.66	1.95 ± 0.79	0.219	1.58 ± 0.61	1.68 ± 0.63	0.111
Second peak to foot-off (I4)	1.14 ± 0.41	1.43 ± 0.71	**0.014**	1.05 ± 0.49	1.41 ± 0.85	**<0.001**
Foot-strike to min (I5)	2.36 ± 0.79	2.42 ± 0.86	0.554	2.44 ± 0.99	2.66 ± 0.90	**0.001**
Min to foot-off (I6)	2.89 ± 1.00	3.30 ± 1.24	**0.009**	2.56 ± 0.98	3.01 ± 1.30	**<0.001**
Foot-strike to foot-off (I7)	5.19 ± 1.62	5.66 ± 1.89	**0.026**	4.89 ± 1.74	5.61 ± 2.01	**<0.001**

**Table 2:** Means and SD for head accelerometer variables with a significant (*p* < 0.05) mixed-design ANOVA result (Supplementary Table S2). Bold *p*-values indicate significant differences between ST and DT conditions after correction for multiple comparisons.

	Fallers			Non-Fallers		
	ST	DT	*p*	ST	DT	*p*
**Fast Fourier Transform (FFT) First Quartile (%)**						
Vertical	45.0 ± 13.0	37.6 ± 10.0	**0.009**	46.4 ± 13.7	39.3 ± 12.9	**<0.001**
AP	50.4 ± 9.9	44.0 ± 7.3	**0.011**	53.5 ± 10.5	49.3 ± 10.4	**<0.001**
ML	56.3 ± 10.1	50.5 ± 10.8	0.065	54.7 ± 12.4	50.6 ± 11.1	0.033
**Ratio of Even to Odd Harmonics**						
Vertical	2.17 ± 0.58	1.99 ± 0.94	0.210	2.17 ± 1.12	1.77 ± 0.92	0.033
AP	1.90 ± 0.78	1.45 ± 0.63	0.033	1.60 ± 0.83	1.50 ± 0.61	0.420
**Maximum Lyapunov Exponent**						
ML	0.24 ± 0.09	0.30 ± 0.10	0.088	0.25 ± 0.09	0.27 ± 0.12	0.391
**Acceleration Descriptive Statistics (g)**						
Superior Max	0.27 ± 0.08	0.33 ± 0.08	**0.001**	0.23 ± 0.07	0.29 ± 0.09	**0.001**
Superior Mean	0.11 ± 0.04	0.13 ± 0.03	**0.002**	0.10 ± 0.03	0.12 ± 0.03	**0.005**
Superior SD	0.07 ± 0.02	0.08 ± 0.02	**0.006**	0.06 ± 0.02	0.08 ± 0.02	**0.005**
Anterior Mean	0.14 ± 0.07	0.11 ± 0.05	0.106	0.15 ± 0.06	0.12 ± 0.06	**0.014**
Right Max	0.27 ± 0.10	0.29 ± 0.10	0.179	0.25 ± 0.12	0.30 ± 0.12	**0.003**
Right Mean	0.11 ± 0.05	0.13 ± 0.05	0.084	0.11 ± 0.05	0.13 ± 0.05	**0.004**
Right SD	0.07 ± 0.02	0.08 ± 0.03	0.151	0.07 ± 0.03	0.08 ± 0.03	**0.002**

**Table 3:** Means and SD for posterior pelvis accelerometer variables with a significant (*p* < 0.05) mixed-design ANOVA result (Supplementary Table S3). Bold *p*-values indicate significant differences between ST and DT conditions after correction for multiple comparisons.

	Fallers			Non-Fallers		
	ST	DT	*p*	ST	DT	*p*
**Fast Fourier Transform (FFT) First Quartile (%)**						
Vertical	32.9 ± 10.6	26.3 ± 9.4	0.014	34.8 ± 10.0	26.5 ± 9.1	**<0.001**
AP	40.7 ± 8.5	37.4 ± 7.8	0.076	43.0 ± 9.8	40.0 ± 7.9	0.072
ML	32.7 ± 11.4	29.5 ± 9.6	0.072	34.1 ± 10.6	29.3 ± 10.3	**0.003**
**Ratio of Even to Odd Harmonics**						
Vertical	2.20 ± 0.84	2.00 ± 0.74	0.151	2.25 ± 0.85	1.94 ± 0.79	0.071
AP	2.11 ± 0.76	1.86 ± 0.77	0.088	2.23 ± 0.86	1.90 ± 0.67	**0.037**
**Maximum Lyapunov Exponent**						
ML	0.28 ± 0.12	0.24 ± 0.10	0.295	0.25 ± 0.11	0.21 ± 0.10	**0.037**
**Acceleration Descriptive Statistics (g)**						
Superior Max	0.32 ± 0.08	0.30 ± 0.09	0.569	0.31 ± 0.10	0.28 ± 0.10	**0.011**
Superior Mean	0.11 ± 0.03	0.09 ± 0.03	**0.013**	0.11 ± 0.03	0.09 ± 0.03	**0.001**
Superior SD	0.08 ± 0.02	0.07 ± 0.02	0.045	0.08 ± 0.02	0.07 ± 0.02	**<0.001**
Inferior Max	0.45 ± 0.09	0.41 ± 0.14	0.029	0.44 ± 0.13	0.37 ± 0.15	**<0.001**
Inferior Mean	0.15 ± 0.03	0.14 ± 0.04	**0.023**	0.16 ± 0.05	0.13 ± 0.05	**<0.001**
Inferior SD	0.12 ± 0.02	0.11 ± 0.03	0.032	0.12 ± 0.03	0.10 ± 0.04	**<0.001**
Anterior Max	0.42 ± 0.12	0.37 ± 0.12	**0.004**	0.48 ± 0.17	0.38 ± 0.14	**<0.001**
Anterior Mean	0.15 ± 0.04	0.13 ± 0.04	**0.020**	0.17 ± 0.06	0.14 ± 0.04	**<0.001**
Anterior SD	0.12 ± 0.03	0.10 ± 0.03	**0.001**	0.13 ± 0.05	0.10 ± 0.04	**<0.001**
Posterior Max	0.31 ± 0.10	0.27 ± 0.08	**0.018**	0.28 ± 0.12	0.25 ± 0.11	**0.028**
Posterior Mean	0.12 ± 0.03	0.10 ± 0.03	**0.004**	0.11 ± 0.05	0.10 ± 0.04	**0.011**
Posterior SD	0.08 ± 0.02	0.07 ± 0.02	**0.015**	0.07 ± 0.03	0.06 ± 0.03	**0.005**
Right Max	0.40 ± 0.11	0.37 ± 0.15	0.053	0.38 ± 0.13	0.31 ± 0.12	**<0.001**
Right Mean	0.13 ± 0.03	0.12 ± 0.04	0.050	0.13 ± 0.04	0.10 ± 0.03	**<0.001**
Right SD	0.11 ± 0.03	0.10 ± 0.05	**0.021**	0.10 ± 0.03	0.08 ± 0.03	**<0.001**
Left Max	0.40 ± 0.08	0.36 ± 0.09	0.068	0.39 ± 0.13	0.33 ± 0.14	**<0.001**
Left Mean	0.13 ± 0.03	0.11 ± 0.03	**0.005**	0.13 ± 0.04	0.10 ± 0.04	**<0.001**
Left SD	0.10 ± 0.02	0.09 ± 0.02	**0.020**	0.10 ± 0.03	0.08 ± 0.03	**<0.001**

**Table 4:** Means and SD for right shank accelerometer variables with a significant (*p* < 0.05) mixed-design ANOVA result (Supplementary Table S4). Bold *p*-values indicate significant differences between ST and DT conditions after correction for multiple comparisons.

	Fallers			Non-Fallers		
	ST	DT	*p*	ST	DT	*p*
**Fast Fourier Transform (FFT) First Quartile (%)**						
Vertical	38.6 ± 11.4	29.9 ± 10.2	**0.006**	39.3 ± 12.7	30.2 ± 10.9	**<0.001**
AP	27.3 ± 8.1	20.7 ± 6.0	**0.005**	29.9 ± 8.8	22.1 ± 7.6	**<0.001**
ML	25.9 ± 7.6	20.0 ± 6.3	**0.002**	28.2 ± 8.0	21.3 ± 6.4	**<0.001**
**Maximum Lyapunov Exponent**						
AP	0.50 ± 0.15	0.43 ± 0.13	0.059	0.48 ± 0.15	0.43 ± 0.15	0.058
**Acceleration Descriptive Statistics (g)**						
Superior Max	0.47 ± 0.18	0.46 ± 0.16	0.762	0.56 ± 0.19	0.48 ± 0.17	**<0.001**
Superior Mean	0.15 ± 0.04	0.14 ± 0.04	0.600	0.18 ± 0.06	0.15 ± 0.05	**0.010**
Superior SD	0.12 ± 0.04	0.12 ± 0.04	0.189	0.15 ± 0.05	0.12 ± 0.05	**<0.001**
Inferior Max	0.74 ± 0.32	0.65 ± 0.27	**0.014**	0.82 ± 0.31	0.65 ± 0.24	**<0.001**
Inferior Mean	0.21 ± 0.08	0.18 ± 0.07	**0.001**	0.22 ± 0.07	0.18 ± 0.07	**<0.001**
Inferior SD	0.20 ± 0.09	0.16 ± 0.07	**0.001**	0.22 ± 0.08	0.17 ± 0.07	**<0.001**
Anterior Max	1.58 ± 0.44	1.32 ± 0.40	**0.004**	1.71 ± 0.58	1.26 ± 0.60	**<0.001**
Anterior Mean	0.40 ± 0.08	0.33 ± 0.08	**0.001**	0.44 ± 0.14	0.32 ± 0.13	**<0.001**
Anterior SD	0.44 ± 0.13	0.34 ± 0.12	**0.001**	0.49 ± 0.18	0.34 ± 0.18	**<0.001**
Posterior Mean	0.29 ± 0.07	0.28 ± 0.06	0.412	0.31 ± 0.09	0.28 ± 0.08	**<0.001**
Right Max	0.57 ± 0.20	0.47 ± 0.16	**0.007**	0.61 ± 0.21	0.51 ± 0.19	**<0.001**
Right Mean	0.17 ± 0.05	0.14 ± 0.05	**0.011**	0.18 ± 0.06	0.15 ± 0.06	**0.001**
Right SD	0.15 ± 0.05	0.12 ± 0.04	**0.002**	0.16 ± 0.06	0.13 ± 0.05	**<0.001**
Left Max	0.71 ± 0.33	0.64 ± 0.27	0.068	0.77 ± 0.31	0.62 ± 0.24	**<0.001**
Left Mean	0.23 ± 0.11	0.20 ± 0.09	**0.019**	0.25 ± 0.10	0.19 ± 0.07	**<0.001**
Left SD	0.22 ± 0.12	0.18 ± 0.09	**0.002**	0.24 ± 0.10	0.18 ± 0.08	**<0.001**

**Table 5:** Means and SD for left shank accelerometer variables with a significant (*p* < 0.05) mixed-design ANOVA result (Supplementary Table S5). Bold *p*-values indicate significant differences between ST and DT conditions after correction for multiple comparisons.

	Fallers			Non-Fallers		
	ST	DT	*p*	ST	DT	*p*
**Fast Fourier Transform (FFT) First Quartile (%)**						
Vertical	34.8 ± 12.9	28.9 ± 11.7	0.046	37.9 ± 12.7	29.6 ± 10.3	**<0.001**
AP	26.4 ± 8.3	20.8 ± 7.0	**0.005**	28.4 ± 8.3	21.6 ± 7.1	**<0.001**
ML	21.5 ± 7.4	17.3 ± 4.9	0.056	25.3 ± 8.6	19.5 ± 7.6	**<0.001**
**Ratio of Even to Odd Harmonics**						
Vertical	1.27 ± 0.43	1.11 ± 0.25	0.056	1.17 ± 0.31	1.22 ± 0.40	0.482
**Maximum Lyapunov Exponent**						
AP	0.48 ± 0.16	0.38 ± 0.16	**0.011**	0.45 ± 0.13	0.43 ± 0.15	0.544
ML	0.38 ± 0.17	0.27 ± 0.14	**0.003**	0.37 ± 0.16	0.30 ± 0.15	**0.010**
**Acceleration Descriptive Statistics (g)**						
Superior Max	0.70 ± 0.34	0.60 ± 0.26	**0.015**	0.71 ± 0.31	0.56 ± 0.26	**<0.001**
Superior Mean	0.20 ± 0.06	0.17 ± 0.05	**0.004**	0.21 ± 0.08	0.17 ± 0.06	**<0.001**
Superior SD	0.19 ± 0.09	0.16 ± 0.07	**0.005**	0.20 ± 0.09	0.15 ± 0.08	**<0.001**
Inferior Max	0.82 ± 0.28	0.75 ± 0.23	**0.027**	0.85 ± 0.28	0.76 ± 0.26	**0.001**
Inferior Mean	0.20 ± 0.06	0.18 ± 0.05	**0.003**	0.22 ± 0.07	0.18 ± 0.06	**<0.001**
Inferior SD	0.21 ± 0.07	0.18 ± 0.06	**0.003**	0.23 ± 0.08	0.18 ± 0.07	**<0.001**
Anterior Max	1.49 ± 0.45	1.22 ± 0.40	**0.001**	1.58 ± 0.41	1.25 ± 0.45	**<0.001**
Anterior Mean	0.42 ± 0.11	0.32 ± 0.10	**<0.001**	0.45 ± 0.14	0.33 ± 0.12	**<0.001**
Anterior SD	0.44 ± 0.15	0.34 ± 0.13	**<0.001**	0.47 ± 0.13	0.34 ± 0.14	**<0.001**
Posterior Mean	0.28 ± 0.06	0.24 ± 0.06	**<0.001**	0.28 ± 0.08	0.26 ± 0.09	**0.008**
Posterior SD	0.27 ± 0.08	0.25 ± 0.07	**0.011**	0.27 ± 0.08	0.27 ± 0.10	0.516
Right Mean	0.21 ± 0.07	0.19 ± 0.05	0.068	0.20 ± 0.05	0.18 ± 0.05	**<0.001**
Right SD	0.22 ± 0.09	0.20 ± 0.07	0.065	0.21 ± 0.06	0.19 ± 0.07	**0.039**
Left Max	0.78 ± 0.33	0.67 ± 0.25	**0.010**	0.82 ± 0.33	0.65 ± 0.29	**<0.001**
Left Mean	0.20 ± 0.07	0.17 ± 0.06	**0.005**	0.23 ± 0.09	0.18 ± 0.07	**<0.001**
Left SD	0.20 ± 0.08	0.16 ± 0.07	**0.004**	0.22 ± 0.10	0.16 ± 0.08	**<0.001**

## Supplementary Materials

The following are available online at http://www.mdpi.com/1424-8220/18/4/1275/s1, Supplementary Tables S1: Mixed-design ANOVA tests results for pressure-sensing insole variables, Table S2: Mixed-design ANOVA tests results for head accelerometer variables, Table S3: Mixed-design ANOVA tests results for posterior pelvis accelerometer variables, Table S4: Mixed-design ANOVA tests results for right shank accelerometer variables, Table S5: Mixed-design ANOVA tests results for left shank accelerometer variables.
