# Supplementary material for: Dual-Task Elderly Gait of Prospective Fallers and Non-Fallers: A Wearable-Sensor Based Analysis
Source: Sensors (Basel). 2018 Apr 21;18(4):1275. doi: 10.3390/s18041275 (PMC5948604; doi:10.3390/s18041275)
Supplement: Supplementary file 1 [file sensors-18-01275-s001.pdf]

Supplementary Tables

Table S1. Mixed-design ANOVA tests results for pressure-sensing insole variables. Bold indicates a significant difference (p < 0.05).

	Mixed-Design ANOVA Analysis		
	**Walking Condition Main Effect**	**Faller/Non-Faller Status Main Effect**	**Interaction Effect**
**CoP Path**			
PD per Stride	**$F(1,73) = 31.166$, $p < 0.001$, $\eta^2 = 0.299$**	$F(1,73) = 0.102$, $p = 0.750$, $\eta^2 = 0.001$	$F(1,73) = 0.318$, $p = 0.575$, $\eta^2 = 0.004$
PD Length (mm)	$F(1,73) = 0.144$, $p = 0.705$, $\eta^2 = 0.002$	$F(1,73) = 0.070$, $p = 0.792$, $\eta^2 = 0.001$	$F(1,73) = 0.191$, $p = 0.664$, $\eta^2 = 0.003$
PD Duration (s)	$F(1,73) = 0.651$, $p = 0.423$, $\eta^2 = 0.009$	$F(1,73) = 0.568$, $p = 0.453$, $\eta^2 = 0.008$	$F(1,73) = 0.611$, $p = 0.437$, $\eta^2 = 0.008$
Medial Deviations per Stride (#)	$F(1,73) = 3.039$, $p = 0.086$, $\eta^2 = 0.040$	$F(1,73) = 0.299$, $p = 0.586$, $\eta^2 = 0.004$	$F(1,73) = 0.043$, $p = 0.836$, $\eta^2 = 0.001$
Medial Deviation Length (mm)	$F(1,73) = 1.303$, $p = 0.257$, $\eta^2 = 0.018$	$F(1,73) = 1.952$, $p = 0.167$, $\eta^2 = 0.026$	$F(1,73) = 1.655$, $p = 0.202$, $\eta^2 = 0.022$
Lateral Deviation Length (mm)	**$F(1,73) = 4.705$, $p = 0.033$, $\eta^2 = 0.061$**	$F(1,73) = 0.899$, $p = 0.346$, $\eta^2 = 0.012$	$F(1,73) = 0.346$, $p = 0.558$, $\eta^2 = 0.005$
ML Deviation Duration (s)	**$F(1,73) = 11.527$, $p = 0.001$, $\eta^2 = 0.136$**	$F(1,73) = 0.023$, $p = 0.881$, $\eta^2 < 0.001$	$F(1,73) = 0.311$, $p = 0.579$, $\eta^2 = 0.004$
Min CoP Vel (m/s)	**$F(1,73) = 35.113$, $p < 0.001$, $\eta^2 = 0.325$**	$F(1,73) = 1.024$, $p = 0.315$, $\eta^2 = 0.014$	$F(1,73) = 0.504$, $p = 0.480$, $\eta^2 = 0.007$
Max CoP Vel (m/s)	$F(1,73) = 2.223$, $p = 0.140$, $\eta^2 = 0.030$	$F(1,73) = 0.023$, $p = 0.881$, $\eta^2 < 0.001$	$F(1,73) = 1.323$, $p = 0.254$, $\eta^2 = 0.018$
Mean CoP Vel (m/s)	**$F(1,73) = 68.784$, $p < 0.001$, $\eta^2 = 0.485$**	$F(1,73) = 0.223$, $p = 0.638$, $\eta^2 = 0.003$	$F(1,73) = 0.939$, $p = 0.336$, $\eta^2 = 0.013$
Median CoP Vel (m/s)	**$F(1,73) = 91.911$, $p < 0.001$, $\eta^2 = 0.557$**	$F(1,73) = 0.209$, $p = 0.649$, $\eta^2 = 0.003$	$F(1,73) = 0.062$, $p = 0.804$, $\eta^2 = 0.001$
**Temporal**			
Cadence (steps/minute)	**$F(1,73) = 75.960$, $p < 0.001$, $\eta^2 = 0.510$**	$F(1,73) = 0.003$, $p = 0.957$, $\eta^2 < 0.001$	$F(1,73) = 1.891$, $p = 0.173$, $\eta^2 = 0.025$
Stride Time (s)	**$F(1,73) = 62.868$, $p < 0.001$, $\eta^2 = 0.463$**	$F(1,73) = 0.058$, $p = 0.810$, $\eta^2 = 0.001$	$F(1,73) = 1.790$, $p = 0.185$, $\eta^2 = 0.024$
Stance Time (s)	**$F(1,73) = 51.289$, $p < 0.001$, $\eta^2 = 0.413$**	$F(1,73) = 0.018$, $p = 0.894$, $\eta^2 < 0.001$	$F(1,73) = 0.764$, $p = 0.385$, $\eta^2 = 0.010$
Swing Time (s)	**$F(1,73) = 43.638$, $p < 0.001$, $\eta^2 = 0.374$**	$F(1,73) = 0.448$, $p = 0.505$, $\eta^2 = 0.006$	$F(1,73) = 1.800$, $p = 0.184$, $\eta^2 = 0.024$
Stride Time CoV	**$F(1,73) = 12.405$, $p = 0.001$, $\eta^2 = 0.145$**	$F(1,73) = 0.901$, $p = 0.346$, $\eta^2 = 0.012$	$F(1,73) = 0.523$, $p = 0.472$, $\eta^2 = 0.0074$
Stance Time CoV	$F(1,73) = 0.127$, $p = 0.723$, $\eta^2 = 0.002$	$F(1,73) = 0.695$, $p = 0.407$, $\eta^2 = 0.009$	$F(1,73) = 1.049$, $p = 0.309$, $\eta^2 = 0.014$
Swing Time CoV	$F(1,73) = 0.194$, $p = 0.661$, $\eta^2 = 0.003$	$F(1,73) = 0.354$, $p = 0.554$, $\eta^2 = 0.005$	$F(1,73) = 0.007$, $p = 0.935$, $\eta^2 < 0.001$
Percent Stance Time (%)	$F(1,73) = 0.123$, $p = 0.727$, $\eta^2 = 0.002$	$F(1,73) = 0.214$, $p = 0.645$, $\eta^2 = 0.003$	$F(1,73) = 0.435$, $p = 0.512$, $\eta^2 = 0.006$
Percent Double-Support Time (%)	$F(1,73) = 0.057$, $p = 0.812$, $\eta^2 = 0.001$	$F(1,73) = 0.168$, $p = 0.683$, $\eta^2 = 0.002$	$F(1,73) = 0.304$, $p = 0.583$, $\eta^2 = 0.004$
Stride Time Symmetry Index	**$F(1,73) = 12.003$, $p = 0.001$, $\eta^2 = 0.141$**	$F(1,73) = 0.005$, $p = 0.942$, $\eta^2 < 0.001$	$F(1,73) = 0.121$, $p = 0.729$, $\eta^2 = 0.002$

**CoP Path Stance Phase CoV**			
CoV AP	**F(1,73) = 21.823, p < 0.001, η^2 = 0.230**	F(1,73) = 0.525, p = 0.471, η^2 = 0.007	**F(1,73) = 9.970, p = 0.002, η^2 = 0.120**
CoV ML	**F(1,73) = 10.331, p = 0.002, η^2 = 0.124**	F(1,73) = 0.131, p = 0.718, η^2 = 0.002	F(1,73) = 0.140, p = 0.709, η^2 = 0.002
**Impulse (Ns/kg)**			
Foot-strike to first peak (I1)	**F(1,73) = 30.524, p < 0.001, η^2 = 0.295**	F(1,73) = 0.107, p = 0.744, η^2 = 0.001	F(1,73) = 1.953, p = 0.167, η^2 = 0.026
First peak to min (I2)	**F(1,73) = 6.078, p = 0.016, η^2 = 0.077**	F(1,73) = 0.726, p = 0.397, η^2 = 0.010	F(1,73) = 1.936, p = 0.168, η^2 = 0.026
Min to second peak (I3)	**F(1,73) = 4.115, p = 0.046, η^2 = 0.053**	F(1,73) = 3.075, p = 0.084, η^2 = 0.040	F(1,73) = 0.050, p = 0.824, η^2 = 0.001
Second peak to foot-off (I4)	**F(1,73) = 22.006, p < 0.001, η^2 = 0.232**	F(1,73) = 0.132, p = 0.717, η^2 = 0.002	F(1,73) = 0.257, p = 0.614, η^2 = 0.004
Foot-strike to min (I5)	**F(1,73) = 5.566, p = 0.021, η^2 = 0.071**	F(1,73) = 0.578, p = 0.450, η^2 = 0.008	F(1,73) = 1.921, p = 0.170, η^2 = 0.026
Min to foot-off (I6)	**F(1,73) = 21.351, p < 0.001, η^2 = 0.226**	F(1,73) = 1.449, p = 0.233, η^2 = 0.019	F(1,73) = 0.060, p = 0.807, η^2 = 0.001
Foot-strike to foot-off (I7)	**F(1,73) = 22.578, p < 0.001, η^2 = 0.236**	F(1,73) = 0.162, p = 0.688, η^2 = 0.002	F(1,73) = 1.037, p = 0.312, η^2 = 0.014

Table S2. Mixed-design ANOVA tests results for head accelerometer variables. Bold indicates a significant difference (p < 0.05).

	**Mixed-Design ANOVA Analysis**		
	**Walking Condition Main Effect**	**Faller/Non-Faller Status Main Effect**	**Interaction Effect**
**FFT First Quartile (%)**			
Vertical	**F(1,73) = 29.151, p < 0.001, η^2 = 0.285**	F(1,73) = 0.334, p = 0.565, η^2 = 0.005	F(1,73) = 0.021, p = 0.884, η^2 < 0.001
AP	**F(1,73) = 21.982, p < 0.001, η^2 = 0.231**	**F(1,73) = 4.217, p = 0.044, η^2 = 0.055**	F(1,73) = 1.027, p = 0.314, η^2 = 0.014
ML	**F(1,73) = 10.335, p = 0.002, η^2 = 0.124**	F(1,73) = 0.126, p = 0.723, η^2 = 0.002	F(1,73) = 0.268, p = 0.607, η^2 = 0.004
**Ratio of Even to Odd Harmonics**			
Vertical	**F(1,73) = 4.297, p = 0.042, η^2 = 0.056**	F(1,73) = 0.384, p = 0.537, η^2 = 0.005	F(1,73) = 0.618, p = 0.434, η^2 = 0.008
AP	**F(1,73) = 7.692, p = 0.007, η^2 = 0.095**	F(1,73) = 0.735, p = 0.394, η^2 = 0.010	F(1,73) = 3.038, p = 0.086, η^2 = 0.040
ML	F(1,73) = 1.104, p = 0.297, η^2 = 0.015	F(1,73) = 1.333, p = 0.252, η^2 = 0.018	F(1,73) = 0.296, p = 0.588, η^2 = 0.004
**Maximum Lyapunov Exponent**			
Vertical	F(1,73) = 2.846, p = 0.096, η^2 = 0.038	F(1,73) = 0.315, p = 0.576, η^2 = 0.004	F(1,73) = 1.237, p = 0.270, η^2 = 0.017
AP	F(1,73) = 0.106, p = 0.746, η^2 = 0.001	F(1,73) = 0.741, p = 0.392, η^2 = 0.010	F(1,73) = 2.190, p = 0.143, η^2 = 0.029
ML	**F(1,73) = 5.331, p = 0.024, η^2 = 0.068**	F(1,73) = 0.182, p = 0.671, η^2 = 0.002	F(1,73) = 1.116, p = 0.294, η^2 = 0.015
**Acceleration Descriptive Statistics (g)**			
Superior Max	**F(1,73) = 29.960, p < 0.001, η^2 = 0.291**	**F(1,73) = 5.376, p =0.023, η^2 = 0.069**	F(1,73) = 0.101, p = 0.751, η^2 = 0.001
Superior Mean	**F(1,73) = 22.368, p < 0.001, η^2 = 0.235**	F(1,73) = 3.131, p = 0.081, η^2 = 0.041	F(1,73) = 0.793, p = 0.376, η^2 = 0.011
Superior SD	**F(1,73) = 18.481, p < 0.001, η^2 = 0.202**	F(1,73) = 3.363, p = 0.071, η^2 = 0.044	F(1,73) = 0.078, p = 0.781, η^2 = 0.001
Inferior Max	F(1,73) = 2.013, p = 0.160, η^2 = 0.027	F(1,73) = 0.043, p = 0.836, η^2 = 0.001	F(1,73) = 0.247, p = 0.620, η^2 = 0.003
Inferior Mean	F(1,73) = 1.931, p = 0.169, η^2 = 0.026	F(1,73) = 0.118, p = 0.732, η^2 = 0.002	F(1,73) = 0.123, p = 0.726, η^2 = 0.002
Inferior SD	F(1,73) = 2.328, p = 0.131, η^2 = 0.031	F(1,73) = 0.019, p = 0.891, η^2 < 0.001	F(1,73) = 0.186, p = 0.668, η^2 = 0.003
Anterior Max	F(1,73) = 0.955, p = 0.332, η^2 = 0.013	F(1,73) = 0.921, p = 0.340, η^2 = 0.012	F(1,73) = 0.123, p = 0.727, η^2 = 0.002
Anterior Mean	**F(1,73) = 7.928, p = 0.006, η^2 = 0.098**	F(1,73) = 1.294, p = 0.259, η^2 = 0.017	F(1,73) = 0.089, p = 0.767, η^2 = 0.001
Anterior SD	F(1,73) = 1.836, p = 0.180, η^2 = 0.025	F(1,73) = 0.958, p = 0.331, η^2 = 0.013	F(1,73) = 0.341, p = 0.561, η^2 = 0.005
Posterior Max	F(1,73) = 0.021, p = 0.885, η^2 < 0.001	F(1,73) = 0.917, p = 0.342, η^2 = 0.012	F(1,73) = 0.063, p = 0.802, η^2 = 0.001
Posterior Mean	F(1,73) = 0.287, p = 0.594, η^2 = 0.004	F(1,73) = 0.323, p = 0.571, η^2 = 0.004	F(1,73) = 0.006, p = 0.939, η^2 < 0.001
Posterior SD	F(1,73) = 0.287, p = 0.594, η^2 = 0.004	F(1,73) = 0.822, p = 0.368, η^2 = 0.011	F(1,73) = 0.002, p = 0.960, η^2 < 0.001
Right Max	**F(1,73) = 8.653, p = 0.004,**	F(1,73) = 0.021, p = 0.884,	F(1,73) = 1.253, p = 0.267,

	**$\eta^2 = 0.106$**	$\eta^2 < 0.001$	$\eta^2 = 0.017$
Right Mean	**$F(1,73) = 10.375$, $p = 0.002$, $\eta^2 = 0.124$**	$F(1,73) = 0.340$, $p = 0.562$, $\eta^2 = 0.005$	$F(1,73) = 0.075$, $p = 0.784$, $\eta^2 = 0.001$
Right SD	**$F(1,73) = 10.907$, $p = 0.001$, $\eta^2 = 0.130$**	$F(1,73) = 0.007$, $p = 0.935$, $\eta^2 < 0.001$	$F(1,73) = 0.842$, $p = 0.362$, $\eta^2 = 0.011$
Left Max	$F(1,73) = 2.022$, $p = 0.159$, $\eta^2 = 0.027$	$F(1,73) = 0.879$, $p = 0.352$, $\eta^2 = 0.012$	$F(1,73) = 2.011$, $p = 0.160$, $\eta^2 = 0.027$
Left Mean	$F(1,73) = 2.989$, $p = 0.088$, $\eta^2 = 0.039$	$F(1,73) = 1.952$, $p = 0.167$, $\eta^2 = 0.026$	$F(1,73) = 3.087$, $p = 0.083$, $\eta^2 = 0.041$
Left SD	$F(1,73) = 0.602$, $p = 0.440$, $\eta^2 = 0.008$	$F(1,73) = 0.453$, $p = 0.503$, $\eta^2 = 0.006$	$F(1,73) = 1.253$, $p = 0.267$, $\eta^2 = 0.017$

Table S3. Mixed-design ANOVA tests results for posterior pelvis accelerometer variables. Bold indicates a significant difference ($p < 0.05$).

	Mixed-Design ANOVA Analysis		
	**Walking Condition Main Effect**	**Faller/Non-Faller Status Main Effect**	**Interaction Effect**
**FFT First Quartile (%)**			
Vertical	**$F(1,71) = 33.338$, $p < 0.001$, $\eta^2 = 0.320$**	$F(1,71) = 0.259$, $p = 0.612$, $\eta^2 = 0.004$	$F(1,71) = 0.422$, $p = 0.518$, $\eta^2 = 0.006$
AP	**$F(1,71) = 8.637$, $p = 0.004$, $\eta^2 = 0.108$**	$F(1,71) = 1.981$, $p = 0.164$, $\eta^2 = 0.027$	$F(1,71) = 0.023$, $p = 0.879$, $\eta^2 < 0.001$
ML	**$F(1,71) = 12.400$, $p = 0.001$, $\eta^2 = 0.149$**	$F(1,71) = 0.076$, $p = 0.784$, $\eta^2 = 0.001$	$F(1,71) = 0.478$, $p = 0.491$, $\eta^2 = 0.007$
**Ratio of Even to Odd Harmonics**			
Vertical	**$F(1,71) = 4.108$, $p = 0.046$, $\eta^2 = 0.055$**	$F(1,71) = 0.001$, $p = 0.971$, $\eta^2 < 0.001$	$F(1,71) = 0.162$, $p = 0.688$, $\eta^2 = 0.002$
AP	**$F(1,71) = 5.850$, $p = 0.018$, $\eta^2 = 0.076$**	$F(1,71) = 0.342$, $p = 0.560$, $\eta^2 = 0.005$	$F(1,71) = 0.084$, $p = 0.773$, $\eta^2 = 0.001$
ML	$F(1,71) = 0.792$, $p = 0.377$, $\eta^2 = 0.011$	$F(1,71) = 0.030$, $p = 0.862$, $\eta^2 < 0.001$	$F(1,71) = 0.004$, $p = 0.953$, $\eta^2 < 0.001$
**Maximum Lyapunov Exponent**			
Vertical	$F(1,71) = 3.627$, $p = 0.061$, $\eta^2 = 0.049$	$F(1,71) = 0.012$, $p = 0.912$, $\eta^2 < 0.001$	$F(1,71) = 0.744$, $p = 0.391$, $\eta^2 = 0.010$
AP	$F(1,71) = 0.073$, $p = 0.789$, $\eta^2 = 0.001$	$F(1,71) = 0.008$, $p = 0.928$, $\eta^2 < 0.001$	$F(1,71) = 0.051$, $p = 0.822$, $\eta^2 = 0.001$
ML	**$F(1,71) = 6.913$, $p = 0.010$, $\eta^2 = 0.089$**	$F(1,71) = 2.688$, $p = 0.106$, $\eta^2 = 0.036$	$F(1,71) = 0.002$, $p = 0.969$, $\eta^2 < 0.001$
**Acceleration Descriptive Statistics (g)**			
Superior Max	**$F(1,71) = 5.609$, $p = 0.021$, $\eta^2 = 0.073$**	$F(1,71) = 0.741$, $p = 0.392$, $\eta^2 = 0.010$	$F(1,71) = 0.864$, $p = 0.356$, $\eta^2 = 0.012$
Superior Mean	**$F(1,71) = 17.158$, $p < 0.001$, $\eta^2 = 0.195$**	$F(1,71) = 0.142$, $p = 0.708$, $\eta^2 = 0.002$	$F(1,71) = 0.863$, $p = 0.356$, $\eta^2 = 0.012$
Superior SD	**$F(1,71) = 18.516$, $p < 0.001$, $\eta^2 = 0.207$**	$F(1,71) = 0.473$, $p = 0.494$, $\eta^2 = 0.007$	$F(1,71) = 0.827$, $p = 0.366$, $\eta^2 = 0.012$
Inferior Max	**$F(1,71) = 21.109$, $p < 0.001$, $\eta^2 = 0.229$**	$F(1,71) = 0.605$, $p = 0.439$, $\eta^2 = 0.008$	$F(1,71) = 1.682$, $p = 0.199$, $\eta^2 = 0.023$
Inferior Mean	**$F(1,71) = 19.490$, $p < 0.001$, $\eta^2 = 0.215$**	$F(1,71) = 0.272$, $p = 0.603$, $\eta^2 = 0.004$	$F(1,71) = 1.404$, $p = 0.240$, $\eta^2 = 0.019$
Inferior SD	**$F(1,71) = 26.319$, $p < 0.001$, $\eta^2 = 0.270$**	$F(1,71) = 0.663$, $p = 0.418$, $\eta^2 = 0.009$	$F(1,71) = 2.666$, $p = 0.107$, $\eta^2 = 0.036$
Anterior Max	**$F(1,71) = 29.255$, $p < 0.001$, $\eta^2 = 0.292$**	$F(1,71) = 1.067$, $p = 0.305$, $\eta^2 = 0.015$	$F(1,71) = 2.602$, $p = 0.111$, $\eta^2 = 0.035$
Anterior Mean	**$F(1,71) = 15.505$, $p < 0.001$, $\eta^2 = 0.179$**	$F(1,71) = 1.203$, $p = 0.276$, $\eta^2 = 0.017$	$F(1,71) = 1.062$, $p = 0.306$, $\eta^2 = 0.015$
Anterior SD	**$F(1,71) = 32.727$, $p < 0.001$, $\eta^2 = 0.316$**	$F(1,71) = 1.405$, $p = 0.240$, $\eta^2 = 0.019$	$F(1,71) = 1.627$, $p = 0.206$, $\eta^2 = 0.022$
Posterior Max	**$F(1,71) = 7.766$, $p = 0.007$, $\eta^2 = 0.099$**	$F(1,71) = 1.163$, $p = 0.284$, $\eta^2 = 0.016$	$F(1,71) = 0.138$, $p = 0.711$, $\eta^2 = 0.002$
Posterior Mean	**$F(1,71) = 9.866$, $p = 0.002$, $\eta^2 = 0.122$**	$F(1,71) = 0.060$, $p = 0.807$, $\eta^2 = 0.001$	$F(1,71) = 0.324$, $p = 0.571$, $\eta^2 = 0.005$
Posterior SD	**$F(1,71) = 10.708$, $p = 0.002$, $\eta^2 = 0.131$**	$F(1,71) = 1.176$, $p = 0.282$, $\eta^2 = 0.016$	$F(1,71) = 0.070$, $p = 0.792$, $\eta^2 = 0.001$
Right Max	**$F(1,71) = 22.869$, $p < 0.001$,**	$F(1,71) = 2.124$, $p = 0.149$,	$F(1,71) = 3.910$, $p = 0.052$,

	**η^2 = 0.244**	η^2 = 0.029	η^2 = 0.052
Right Mean	**F(1,71) = 21.995, *p* < 0.001, η^2 = 0.237**	F(1,71) = 1.866, *p* = 0.176, η^2 = 0.026	F(1,71) = 2.833, *p* = 0.097, η^2 = 0.038
Right SD	**F(1,71) = 27.996, *p* < 0.001, η^2 = 0.283**	F(1,71) = 2.958, *p* = 0.090, η^2 = 0.040	F(1,71) = 3.506, *p* = 0.065, η^2 = 0.047
Left Max	**F(1,71) = 11.974, *p* = 0.001, η^2 = 0.144**	F(1,71) = 0.319, *p* = 0.574, η^2 = 0.004	F(1,71) = 0.686, *p* = 0.410, η^2 = 0.010
Left Mean	**F(1,71) = 22.865, *p* < 0.001, η^2 = 0.244**	F(1,71) = 0.001, *p* = 0.972, η^2 < 0.001	F(1,71) = 0.841, *p* = 0.362, η^2 = 0.012
Left SD	**F(1,71) = 26.720, *p* < 0.001, η^2 = 0.273**	F(1,71) = 0.118, *p* = 0.733, η^2 = 0.002	F(1,71) = 0.992, *p* = 0.323, η^2 = 0.014

Table S4. Mixed-design ANOVA tests results for right shank accelerometer variables. Bold indicates a significant difference (p < 0.05).

	**Mixed-Design ANOVA Analysis**		
	**Walking Condition Main Effect**	**Faller/Non-Faller Status Main Effect**	**Interaction Effect**
**FFT First Quartile (%)**			
Vertical	**$F(1,73) = 30.897$, $p < 0.001$, $\eta^2 = 0.297$**	$F(1,73) = 0.044$, $p = 0.834$, $\eta^2 = 0.001$	$F(1,73) = 0.022$, $p = 0.884$, $\eta^2 < 0.001$
AP	**$F(1,73) = 42.588$, $p < 0.001$, $\eta^2 = 0.368$**	$F(1,73) = 1.753$, $p = 0.190$, $\eta^2 = 0.023$	$F(1,73) = 0.301$, $p = 0.585$, $\eta^2 = 0.004$
ML	**$F(1,73) = 47.478$, $p < 0.001$, $\eta^2 = 0.394$**	$F(1,73) = 1.491$, $p = 0.226$, $\eta^2 = 0.020$	$F(1,73) = 0.287$, $p = 0.594$, $\eta^2 = 0.004$
**Ratio of Even to Odd Harmonics**			
Vertical	$F(1,73) = 1.463$, $p = 0.230$, $\eta^2 = 0.020$	$F(1,73) = 0.001$, $p = 0.975$, $\eta^2 < 0.001$	$F(1,73) = 0.053$, $p = 0.818$, $\eta^2 = 0.001$
AP	$F(1,73) = 0.021$, $p = 0.885$, $\eta^2 < 0.001$	$F(1,73) = 0.001$, $p = 0.975$, $\eta^2 < 0.001$	$F(1,73) = 0.058$, $p = 0.810$, $\eta^2 = 0.001$
ML	$F(1,73) = 3.334$, $p = 0.072$, $\eta^2 = 0.044$	$F(1,73) = 1.352$, $p = 0.249$, $\eta^2 = 0.018$	$F(1,73) = 2.087$, $p = 0.153$, $\eta^2 = 0.028$
**Maximum Lyapunov Exponent**			
Vertical	$F(1,73) = 0.202$, $p = 0.654$, $\eta^2 = 0.003$	$F(1,73) = 0.634$, $p = 0.428$, $\eta^2 = 0.009$	$F(1,73) = 0.059$, $p = 0.809$, $\eta^2 = 0.001$
AP	**$F(1,73) = 7.656$, $p = 0.007$, $\eta^2 = 0.095$**	$F(1,73) = 0.156$, $p = 0.694$, $\eta^2 = 0.002$	$F(1,73) = 0.324$, $p = 0.571$, $\eta^2 = 0.004$
ML	$F(1,73) = 3.249$, $p = 0.076$, $\eta^2 = 0.043$	$F(1,73) = 0.324$, $p = 0.571$, $\eta^2 = 0.004$	$F(1,73) = 0.799$, $p = 0.374$, $\eta^2 = 0.011$
**Acceleration Descriptive Statistics (g)**			
Superior Max	**$F(1,73) = 8.303$, $p = 0.005$, $\eta^2 = 0.102$**	$F(1,73) = 1.820$, $p = 0.182$, $\eta^2 = 0.024$	**$F(1,73) = 6.279$, $p = 0.014$, $\eta^2 = 0.079$**
Superior Mean	**$F(1,73) = 5.796$, $p = 0.019$, $\eta^2 = 0.074$**	$F(1,73) = 2.956$, $p = 0.090$, $\eta^2 = 0.039$	$F(1,73) = 1.677$, $p = 0.199$, $\eta^2 = 0.022$
Superior SD	**$F(1,73) = 13.103$, $p = 0.001$, $\eta^2 = 0.152$**	$F(1,73) = 1.753$, $p = 0.190$, $\eta^2 = 0.023$	**$F(1,73) = 4.513$, $p = 0.037$, $\eta^2 = 0.058$**
Inferior Max	**$F(1,73) = 27.457$, $p < 0.001$, $\eta^2 = 0.273$**	$F(1,73) = 0.488$, $p = 0.487$, $\eta^2 = 0.007$	$F(1,73) = 2.097$, $p = 0.152$, $\eta^2 = 0.028$
Inferior Mean	**$F(1,73) = 30.777$, $p < 0.001$, $\eta^2 = 0.297$**	$F(1,73) = 0.336$, $p = 0.564$, $\eta^2 = 0.005$	$F(1,73) = 0.393$, $p = 0.533$, $\eta^2 = 0.005$
Inferior SD	**$F(1,73) = 42.857$, $p < 0.001$, $\eta^2 = 0.370$**	$F(1,73) = 0.404$, $p = 0.527$, $\eta^2 = 0.006$	$F(1,73) = 1.987$, $p = 0.163$, $\eta^2 = 0.027$
Anterior Max	**$F(1,73) = 39.795$, $p < 0.001$, $\eta^2 = 0.353$**	$F(1,73) = 0.131$, $p = 0.718$, $\eta^2 = 0.002$	$F(1,73) = 2.950$, $p = 0.090$, $\eta^2 = 0.039$
Anterior Mean	**$F(1,73) = 43.567$, $p < 0.001$, $\eta^2 = 0.374$**	$F(1,73) = 0.435$, $p = 0.512$, $\eta^2 = 0.006$	$F(1,73) = 2.218$, $p = 0.141$, $\eta^2 = 0.029$
Anterior SD	**$F(1,73) = 45.927$, $p < 0.001$, $\eta^2 = 0.386$**	$F(1,73) = 0.691$, $p = 0.409$, $\eta^2 = 0.009$	$F(1,73) = 2.405$, $p = 0.125$, $\eta^2 = 0.032$
Posterior Max	$F(1,73) = 0.384$, $p = 0.537$, $\eta^2 = 0.005$	$F(1,73) = 0.210$, $p = 0.648$, $\eta^2 = 0.003$	$F(1,73) = 0.265$, $p = 0.608$, $\eta^2 = 0.004$
Posterior Mean	**$F(1,73) = 10.992$, $p = 0.001$, $\eta^2 = 0.131$**	$F(1,73) = 0.389$, $p = 0.535$, $\eta^2 = 0.005$	$F(1,73) = 2.896$, $p = 0.093$, $\eta^2 = 0.038$
Posterior SD	$F(1,73) = 1.438$, $p = 0.234$, $\eta^2 = 0.019$	$F(1,73) = 0.005$, $p = 0.943$, $\eta^2 < 0.001$	$F(1,73) = 0.034$, $p = 0.854$, $\eta^2 < 0.001$
Right Max	**$F(1,73) = 27.236$, $p < 0.001$,**	$F(1,73) = 0.748$, $p = 0.390$,	$F(1,73) < 0.001$, $p = 0.987$,

	**η^2 = 0.272**	η^2 = 0.010	η^2 <0.001
Right Mean	**F(1,73) = 19.773, *p* < 0.001, η^2 = 0.213**	F(1,73) = 1.263, *p* = 0.265, η^2 = 0.017	F(1,73) = 0.075, *p* = 0.786, η^2 = 0.001
Right SD	**F(1,73) = 35.211, *p* < 0.001, η^2 = 0.325**	F(1,73) = 1.197, *p* = 0.278, η^2 = 0.016	F(1,73) = 0.137, *p* = 0.712, η^2 = 0.002
Left Max	**F(1,73) = 19.078, *p* < 0.001, η^2 = 0.207**	F(1,73) = 0.142, *p* = 0.707, η^2 = 0.002	F(1,73) = 3.014, *p* = 0.087, η^2 = 0.040
Left Mean	**F(1,73) = 26.078, *p* < 0.001, η^2 = 0.263**	F(1,73) = 0.157, *p* = 0.693, η^2 = 0.002	F(1,73) = 3.015, *p* = 0.087, η^2 = 0.040
Left SD	**F(1,73) = 32.828, *p* < 0.001, η^2 = 0.310**	F(1,73) = 0.032, *p* = 0.859, η^2 < 0.001	F(1,73) = 1.906, *p* = 0.172, η^2 = 0.025

Table S5. Mixed-design ANOVA tests results for left shank accelerometer variables. Bold indicates a significant difference (p < 0.05).

	**Mixed-Design ANOVA Analysis**		
	**Walking Condition Main Effect**	**Faller/Non-Faller Status Main Effect**	**Interaction Effect**
**FFT First Quartile (%)**			
Vertical	**$F(1,72) = 20.535$, $p < 0.001$, $\eta^2 = 0.222$**	$F(1,72) = 0.623$, $p = 0.433$, $\eta^2 = 0.009$	$F(1,72) = 0.536$, $p = 0.467$, $\eta^2 = 0.007$
AP	**$F(1,72) = 36.656$, $p < 0.001$, $\eta^2 = 0.337$**	$F(1,72) = 0.846$, $p = 0.361$, $\eta^2 = 0.012$	$F(1,72) = 0.373$, $p = 0.543$, $\eta^2 = 0.005$
ML	**$F(1,72) = 21.428$, $p < 0.001$, $\eta^2 = 0.229$**	**$F(1,72) = 4.360$, $p = 0.040$, $\eta^2 = 0.057$**	$F(1,72) = 0.619$, $p = 0.434$, $\eta^2 = 0.009$
**Ratio of Even to Odd Harmonics**			
Vertical	$F(1,72) = 1.492$, $p = 0.226$, $\eta^2 = 0.020$	$F(1,72) = 0.010$, $p = 0.921$, $\eta^2 < 0.001$	**$F(1,72) = 4.279$, $p = 0.042$, $\eta^2 = 0.056$**
AP	$F(1,72) = 0.082$, $p = 0.775$, $\eta^2 = 0.001$	$F(1,72) = 0.006$, $p = 0.940$, $\eta^2 < 0.001$	$F(1,72) = 0.593$, $p = 0.444$, $\eta^2 = 0.008$
ML	$F(1,72) = 0.003$, $p = 0.958$, $\eta^2 < 0.001$	$F(1,72) = 0.305$, $p = 0.583$, $\eta^2 = 0.004$	$F(1,72) = 0.719$, $p = 0.399$, $\eta^2 = 0.010$
**Maximum Lyapunov Exponent**			
Vertical	$F(1,72) = 0.088$, $p = 0.768$, $\eta^2 = 0.001$	$F(1,72) = 0.464$, $p = 0.498$, $\eta^2 = 0.006$	$F(1,72) = 0.207$, $p = 0.651$, $\eta^2 = 0.003$
AP	**$F(1,72) = 8.853$, $p = 0.004$, $\eta^2 = 0.109$**	$F(1,72) = 0.206$, $p = 0.651$, $\eta^2 = 0.003$	$F(1,72) = 3.730$, $p = 0.057$, $\eta^2 = 0.049$
ML	**$F(1,72) = 18.213$, $p < 0.001$, $\eta^2 = 0.202$**	$F(1,72) = 0.156$, $p = 0.694$, $\eta^2 = 0.002$	$F(1,72) = 0.853$, $p = 0.359$, $\eta^2 = 0.012$
**Acceleration Descriptive Statistics (g)**			
Superior Max	**$F(1,72) = 27.371$, $p < 0.001$, $\eta^2 = 0.275$**	$F(1,72) = 0.068$, $p = 0.795$, $\eta^2 = 0.001$	$F(1,72) = 0.865$, $p = 0.355$, $\eta^2 = 0.012$
Superior Mean	**$F(1,72) = 33.402$, $p < 0.001$, $\eta^2 = 0.317$**	$F(1,72) = 0.259$, $p = 0.612$, $\eta^2 = 0.004$	$F(1,72) = 2.604$, $p = 0.111$, $\eta^2 = 0.035$
Superior SD	**$F(1,72) = 35.620$, $p < 0.001$, $\eta^2 = 0.331$**	$F(1,72) = 0.052$, $p = 0.820$, $\eta^2 = 0.001$	$F(1,72) = 1.000$, $p = 0.321$, $\eta^2 = 0.014$
Inferior Max	**$F(1,72) = 15.530$, $p < 0.001$, $\eta^2 = 0.177$**	$F(1,72) = 0.105$, $p = 0.746$, $\eta^2 = 0.001$	$F(1,72) = 0.353$, $p = 0.554$, $\eta^2 = 0.005$
Inferior Mean	**$F(1,72) = 28.405$, $p < 0.001$, $\eta^2 = 0.283$**	$F(1,72) = 0.874$, $p = 0.353$, $\eta^2 = 0.012$	$F(1,72) = 1.235$, $p = 0.270$, $\eta^2 = 0.017$
Inferior SD	**$F(1,72) = 25.728$, $p < 0.001$, $\eta^2 = 0.263$**	$F(1,72) = 0.257$, $p = 0.614$, $\eta^2 = 0.004$	$F(1,72) = 0.877$, $p = 0.352$, $\eta^2 = 0.012$
Anterior Max	**$F(1,72) = 43.355$, $p < 0.001$, $\eta^2 = 0.376$**	$F(1,72) = 0.493$, $p = 0.485$, $\eta^2 = 0.007$	$F(1,72) = 0.540$, $p = 0.465$, $\eta^2 = 0.007$
Anterior Mean	**$F(1,72) = 46.233$, $p < 0.001$, $\eta^2 = 0.391$**	$F(1,72) = 0.714$, $p = 0.401$, $\eta^2 = 0.010$	$F(1,72) = 0.290$, $p = 0.592$, $\eta^2 = 0.004$
Anterior SD	**$F(1,72) = 52.633$, $p < 0.001$, $\eta^2 = 0.422$**	$F(1,72) = 0.263$, $p = 0.610$, $\eta^2 = 0.004$	$F(1,72) = 1.052$, $p = 0.309$, $\eta^2 = 0.014$
Posterior Max	$F(1,72) = 0.474$, $p = 0.493$, $\eta^2 = 0.007$	$F(1,72) = 0.290$, $p = 0.592$, $\eta^2 = 0.004$	$F(1,72) = 1.918$, $p = 0.170$, $\eta^2 = 0.026$
Posterior Mean	**$F(1,72) = 19.140$, $p < 0.001$, $\eta^2 = 0.210$**	$F(1,72) = 0.985$, $p = 0.324$, $\eta^2 = 0.013$	$F(1,72) = 1.246$, $p = 0.268$, $\eta^2 = 0.017$
Posterior SD	**$F(1,72) = 5.384$, $p = 0.023$, $\eta^2 = 0.070$**	$F(1,72) = 0.481$, $p = 0.490$, $\eta^2 = 0.007$	$F(1,72) = 2.833$, $p = 0.097$, $\eta^2 = 0.038$
Right Max	$F(1,72) = 3.181$, $p = 0.079$,	$F(1,72) = 0.002$, $p = 0.967$,	$F(1,72) = 0.108$, $p = 0.743$,

	$\eta^2 = 0.042$	$\eta^2 < 0.001$	$\eta^2 = 0.002$
Right Mean	**F(1,72) = 17.639, *p* < 0.001, η^2 = 0.197**	F(1,72) = 0.118, *p* = 0.732, $\eta^2 = 0.002$	F(1,72) = 0.019, *p* = 0.891, $\eta^2 < 0.001$
Right SD	**F(1,72) = 8.560, *p* = 0.005, η^2 = 0.106**	F(1,72) = 0.483, *p* = 0.489, $\eta^2 = 0.007$	F(1,72) = 0.102, *p* = 0.751, $\eta^2 = 0.001$
Left Max	**F(1,72) = 23.183, *p* < 0.001, η^2 = 0.244**	F(1,72) = 0.014, *p* = 0.905, $\eta^2 < 0.001$	F(1,72) = 1.351, *p* = 0.249, $\eta^2 = 0.018$
Left Mean	**F(1,72) = 25.453, *p* < 0.001, η^2 = 0.261**	F(1,72) = 0.886, *p* = 0.350, $\eta^2 = 0.012$	F(1,72) = 1.577, *p* = 0.213, $\eta^2 = 0.021$
Left SD	**F(1,72) = 25.796, *p* < 0.001, η^2 = 0.264**	F(1,72) = 0.147, *p* = 0.703, $\eta^2 = 0.002$	F(1,72) = 1.434, *p* = 0.235, $\eta^2 = 0.020$
